# Severe Cutaneous Adverse Reactions: The Pharmacogenomics from Research to Clinical Implementation

**DOI:** 10.3390/ijms17111890

**Published:** 2016-11-15

**Authors:** Shih-Chi Su, Shuen-Iu Hung, Wen-Lang Fan, Ro-Lan Dao, Wen-Hung Chung

**Affiliations:** 1 Whole-Genome Research Core Laboratory of Human Diseases, Chang Gung Memorial Hospital, Keelung 204, Taiwan; ssu1@cgmh.org.tw (S.-C.S.); alangfan@gmail.com (W.-L.F.); 2 Department of Dermatology, Drug Hypersensitivity Clinical and Research Center, Chang Gung Memorial Hospital, Taipei, Linkou and Keelung 105, Taiwan; 3 Institute of Pharmacology, School of Medicine, Infection and Immunity Research Center, National Yang-Ming University, Taipei 112, Taiwan; sihung@ym.edu.tw (S.-I.H.); laura44@ms10.hinet.net (R.-L.D.); 4 College of Medicine, Chang Gung University, Taoyuan 333, Taiwan

**Keywords:** severe cutaneous adverse reactions, pharmacogenomics, clinical implementation

## Abstract

Severe cutaneous adverse reactions (SCARs), previously thought to be idiosyncratic or unpredictable, are a deadly form of adverse drug reactions with skin manifestations. Current pharmacogenomic studies of SCARs have made important strides, as the prevention of SCARs, to some extent, appears attainable with the identification of genetic variants for genes encoding drug-metabolizing enzymes and human leukocyte antigens (HLAs). Despite the improvement of incidence, a treatment guideline for this devastating condition is still unavailable, highlighting the inadequacy of contemporary accepted therapeutic interventions. As such, prompt withdrawal of causative drugs is believed to be a priority of patient management. In this review, we discuss recent cutting-edge findings concerning the discovery of biomarkers for SCARs and their clinical utilities in the better prediction and early diagnosis of this disease. The knowledge compiled herein provides clues for future investigations on deciphering additional genetic markers for SCARs and the design of clinical trials for the prospective identification of subjects at genetic risk for this condition, ultimately personalizing the medicine.

## 1. Introduction

Severe cutaneous adverse drug reactions (SCARs) are potentially lethal events that unexpectedly occur in 2%–3% hospitalized patients [1] upon drug administration and usually result in lifelong sequela. The spectrum of cutaneous adverse drug reactions includes milder forms, such as urticarial and maculopapular exanthema (MPE), and severer types, such as acute generalized exanthematous pustulosis (AGEP), Stevens–Johnson syndrome (SJS), toxic epidermal necrolysis (TEN), and drug reactions with eosinophilia and systemic symptoms (DRESS)/hypersensitivity syndrome (HSS). Although presenting a low incidence, SCARs account for a high mortality from 10% to 40% and frequently incur disability [2,3].

Although there seem to be additional factors that may cause SCARs (such as viral infection and defects in drug metabolism/clearance) [4,5,6], mounting evidence has indicated that many cases of SCARs are specific immune reactions where interactions between human leukocyte antigen (HLA) molecules and particular drugs play an important role in the activation of immune cells (predominantly T lymphocytes) in defined populations [4,7,8,9]. Because drugs are usually too small to potentially induce an immunogenic response, several mechanistic hypotheses including the hapten/prohapten, pharmacological interaction with immune receptors (p–i), and altered repertoire models have been proposed to explain how small compounds are recognized by T cells in an HLA-dependent manner [10,11]. In brief, the hapten/prohapten model delineates that the drug or its metabolite (hapten/prohapten) reacts with a self-protein through covalent binding to produce a haptenated, de novo product [12,13], while the p–i model involves a noncovalent, labile interaction of the drug, with the HLA receptor at the cell surface independent of antigen processing or cellular metabolism [14]. Another hypothesis, the altered repertoire model, postulates that the drugs or its metabolites can bind noncovalently within the pocket of the peptide binding groove of certain HLA molecules, potentiating a new repertoire of endogenous self-peptides to be bound and presented [15,16,17]. Upon the activation of T lymphocytes, multiple immunological and cytotoxic signals are triggered to mediate the damages in skin lesions and the subsequent exacerbation of the disease [18]. In this review, we highlight current cutting-edge findings on the identification of genetic markers for SCARs and their clinical utilities in better prediction and early diagnosis of this disease. In addition, the clinical implications of the immunological and cytotoxic mediators of SCARs, with a focus on SJS/TEN, and DRESS, in the expeditious determination of causative drug(s) and therapeutic interventions are discussed.

## 2. Genetic Susceptibility to SCARs

Current advancements in pharmacogenomic studies have extended our understanding on the genetic basis of SCARs. The relationship between HLA alleles and drug-induced SJS/TEN was first demonstrated in cases of sulfonamide- and oxicam-related TEN [19]. The biological function of HLAs is to present antigens to the T cell receptor (TCR) and then elicit specific T cell-dependent immune responses, which is largely correlated with the pathogenesis of SCARs. It has become clear now that HLA associations with SCARs are often drug- and ethnicity-specific. Our group has previously revealed a strong association of carbamazepine (CBZ)-induced SJS/TEN with the HLA-B*15:02 allele among Han Chinese in Taiwan [20]. This association was also validated in many populations, especially in geographical regions of South-East Asia, such as Hong Kong [21], Malaysia [22], and Thailand [23]. The US Food and Drug Administration (FDA), thus, has recommended genetic testing for all new users of CBZ whose ancestries have high allele frequency of HLA-B*15:02. However, due to different frequencies of HLA-B*15:02 among distinct populations, this genetic association with CBZ-induced SJS was relatively weak in Indians [24] and not observed in Korean, Japanese, and European groups [25,26,27]. Instead of HLA-B*15:02, CBZ-induced hypersensitivity reactions were found to associated with HLA-A*31:01 in European [28] and Japanese populations [29].

Apart from the ethnical specificity, the genetic predispositions to SCARs appear to be attributed largely by the nature of the offending drugs. Instead of HLA-B*15:02, we have shown that HLA-B*58:01 is strongly linked to allopurinol-induced SJS/TEN [30]. Unlike the scenario in CBZ–SJS/TEN, the strong correlation of HLA-B*58:01 with allopurinol-induced SJS/TEN was fairly universal, as it was successfully replicated in not only other Southeast Asians [31] but also in Japanese patients [27] and patients of European origin [32].

Despite the ethnical differences in HLA allele frequencies, as well as variations in sample sizes and clinical classification of the enrolled cases, other HLA-drug associations have been reported in SCARs. These include HLA-B*13:01 with dapsone [33], HLA-A*31:01, and HLA-B*15:11 with CBZ [28,29,34,35], HLA-B*15:02 with phenytoin [23,36], HLA-B*57:01 with abacavir [37], HLA-B*38 with sulfamethoxazole or lamotrigine [32], HLA-B*73 with oxicam [32], and HLA-B*59:01 with methazolamide [38]. A summary of genetic relationships between various HLA allotypes and drug-mediated SCARs is shown in Table 1.

In addition to the immune-related mechanisms, drug metabolism has been found to play a role in the pathogenesis of SCAR. A notable example is the finding that genetic variants of *cytochrome P450 family 2 subfamily C member 9* (*CYP2C9*), encoding an enzyme responsible for metabolic clearance of phenytoin [39], are strongly associated with phenytoin-induced SCARs [5]. Conceivably, impaired clearance of medications derived from either genetic variations or renal insufficiency [6] can result in sustained high levels of culprit drugs or their active metabolite, ultimately leveraging the risk of SCARs. The information obtained from these pharmacogenomic studies is highly beneficial for lowering the incidence of SCARs and ultimately generating genetic databases that allow prescriptions to be tailored to an individual’s genetic risk.

## 3. Clinical Implementation of Genetic Screening to Prevent SCARs

**Figure 1:**
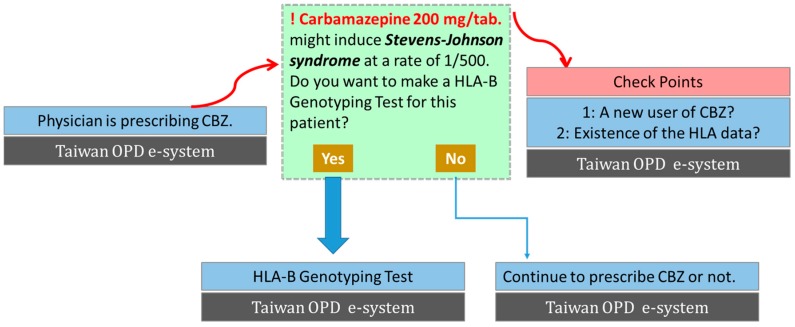
Flowchart of the risky allele screening prior to prescription, an electronic system alert example of carbamazepine (CBZ) prescription at medical centers in Taiwan. HLA: human leukocyte antigen; OPD: out-patient department.

The application of genetic screening for adverse drug reactions would have high utility for those conditions that are prevalent, severe, and associated with genetic markers that exhibit high sensitivity and specificity. As such, genotyping HLAs has been useful in screening for populations at risk for SCARs and excluding them from prescribing certain drugs. The translation of HLA-B*15:02 and CBZ-induced SJS/TEN from discovery to a guideline-based test used routinely in Taiwan is a notable example (Figure 1). Based on the discovery of the strong genetic association, the Taiwan and US Food and Drug Administrations have relabeled the drug information of CBZ and recommended a genetic screening of HLA-B*15:02 in certain Asian groups before use of CBZ in 2007. In 2010, the screening of HLA-B*1502, covered by the Bureau of National Health Insurance (BNHI), was approved as a guideline-based test for patients before the first administration of CBZ in Taiwan. A prospective screening via HLA-B*15:02 genotyping before CBZ treatment was demonstrated to reduce the incidence of CBZ-induced SCARs, although rashes and other adverse reactions occurred as well [50]. Another similar observation where screening of HLA-B*15:02 was relevant to the prevention of CBZ-induced SJS/TEN was obtained in Hong Kong [51]. Nowadays, such testing for preventing CBZ-induced SCARs has been implemented in Taiwan, Hong Kong, Singapore, and many medical centers in Thailand and Mainland China.

Another profound example of the translation of pharmacogenomics findings to clinical utility is the use of HLA-B*58:01 genotyping to prevent allopurinol-induced SCARs. Previously, the annual incidence of allopurinol hypersensitivity rose significantly in Taiwan [52]. Based on the finding that HLA-B*58:01 is associated with allopurinol-induced hypersensitivity universally, the American College of Rheumatology (ACR) guidelines for management of gout recommended HLA-B*58:01 testing prior to the allopurinol administration in 2012 [53]. Recently, a prospective screening for the HLA-B*58:01 allele to identify individuals at risk of SCARs induced by allopurinol treatment was completed in Taiwan [54]. In this trial, genotyping HLA-B*58:01 prior to the use of allopurinol significantly decreased the incidence of allopurinol-induced SCARs. To date, testing for HLA-B*58:01 has been provided in many medical centers in Taiwan, Hong Kong, Thailand, and Mainland China and has appeared beneficial for subjects at risk of allopurinol-associated fatal hypersensitivity reactions.

In addition, HLA-B*57:01 screening prior to abacavir treatment has been widely implemented in routine clinical practice and is part of the US FDA and international human immunodeficiency virus (HIV) treatment guidelines [55]. A clinical trial that was performed to evaluate the clinical utility of pharmacogenetic testing for HLA-B*57:01 on white populations at risk of abacavir-induced hypersensitivity has demonstrated a predictive value [49]. Other ongoing trials aiming to test the preventive power of genetic markers on SCARs include the prospective screening of HLA-B*13:01 for dapsone-induced hypersensitivity in China and Indonesia as well as of CYP2C9*3 and other related HLA alleles for phenytoin-induced SCARs in Taiwan.

## 4. Causative Drug(s) Identification

Although the discovery of the genetic markers has improved the incidence of SCARs, its treatment remains ineffective and only supportive. As such, prompt withdrawal of causative drugs is believed to be a priority of patient management. Until now, more than 100 medications have been associated with this clinical entity [56], among which allopurinol, aromatic anticonvulsants, sulfonamide antibiotics, oxicam nonsteroidal anti-inflammatory drugs, and nevirapine exhibit a high relative risk [57,58,59,60,61]. Patients with SCARs are often exposed simultaneously to a few potentially culprit drugs, thereby making the assessment of drug causality a real challenge. Several in vitro methods have thus emerged to address this issue [62,63]. Among them, the lymphocyte transformation test (LTT) is a reproducible test revealing a sensitization of T cells to a certain drug by an enhanced proliferative response of peripheral blood mononuclear cells (PBMCs) [64]. Although used as a standard technique in the diagnosis of T cell-mediated hypersensitivity reactions for decades, false positives may occur in patients showing elevated PBMC proliferation in the LTT to drugs they have tolerated [65]. In addition, the LTT is often negative, as its sensitivity varies among studies that evaluate diverse drugs and clinical phenotypes [66].

Many cytotoxic assays as an alternative nonradioactive approach, which may be more appropriate for routine testing than a proliferation-based LTT, have been developed, including the enzyme-linked immunospot (ELISpot) assay, intracellular cytokine staining, and the enzyme-linked immunosorbent assay (ELISA) for secretion of cytotoxic mediators. Similar to the LTT, these assays measure the production and release of a target cytokine(s)/cytotoxic protein(s) by a population of T cells with exposure to pharmacological concentrations of the suspected drug or drug metabolite. In the case of SJS/TEN, many “danger signals” are triggered to mediate the damages to keratinocytes in skin lesions [18]. These toxic signals, including inflammatory cytokines, chemokines–chemokine receptors, Fas–Fas ligand (FasL), perforin, granzyme B, and granulysin, were commonly utilized as biomarkers in in vitro assays mentioned above to monitor drug-specific T cell activation for causative drug identification.

Of note, granulysin, a cytotoxic protein excreted by cluster of differentiation (CD) 8^+^ T cells and CD56^+^ natural killer (NK) cells, was previously identified in our genome-wide gene expression profiling of SJS blister cells and found to be essential in the immunopathgenesis of SJS [67]. Granulysin levels were quantitatively associated with the disease severity of SJS/TEN [67] and clinically relevant to DRESS [68,69]. For these particular SCARs, measurement of granulysin using the ELISpot, intracellular cytokine staining, and ELISA may be useful to determine the causative drug, which would be another instance of successful translation from the findings of SCAR research to clinical settings. Not only for culprit drug identification, currently, a rapid immunochromatographic test for serum granulysin has exhibited predictability in the early stage of SJS/TEN 2–4 days prior to typical mucosal and cutaneous symptoms, revealing a usefulness of granulysin in the early diagnosis of SCARs.

## 5. Conclusions and Future Perspectives

In spite of their rarity, SCARs have a huge impact on public health due to high death rates. This clinical entity often leads to a lasting disability of patients and restrains the physicians from prescribing medications that are commonly administered in clinical practice. Encouragingly, current studies have significantly leveraged our understanding in the pathobiology of SCARs. Major advances, such as the discovery of genetic predisposing factors, the clarification of HLA–drug–TCR interactions, and the identification of granulysin as a key mediator of cytotoxic T lymphocytes in SCARs, have been commonly implemented in the clinic, providing us with a sound foundation for disease prevention and early diagnosis. However, there is unfortunately no treatment guideline, highlighting the inadequacy of current accepted regimens [70]. In addition to therapeutic approaches directed against the Fas–FasL interaction [71] or the tumor necrosis factor (TNF)-α pathway [72,73,74], systemic corticosteroids have been the mainstay of SCAR therapies for a long time, which may be largely attributed by the notion that the immunopathogenesis of SCARs involves many cytotoxic and inflammatory mediators. In this regard, reagents antagonizing the effects of granulysin or TCR engagement may be of greater therapeutic value and become an unmet need. Moreover, one additional limitation of implementing specific HLA typing on the prevention of SCARs that needs to be taken into consideration is that, when this type of haplotype screening is applied, a number of patients do avoid experiencing a SCAR, but some patients who could have safely used the drug are denied it. The evaluation for the acceptability and the cost/risks of such a strategy, therefore, will be helpful in addressing this issue [75,76,77]. Taken together, further studies or clinical trials on therapeutic aspects and risk factors are needed to develop more options on disease prevention and management and to achieve a better outcome in unfortunate people who suffer from this deadly adverse drug reaction.

## Figures and Tables

**Table 1:** Ethnicity- and phenotype-specific associations of human leukocyte antigens (HLAs) with severe cutaneous adverse reactions (SCARs).

Causative Drug	SCAR Type	HLA Allele	Ancestry	Region	Reference
Allopurinol	SJS/TEN/DRESS	B*58:01	Han Chinese	Taiwan	[30]
			Caucasian	Europe	[32]
			Thai	Thailand	[31]
			Japanese	Japan	[27]
	Drug eruption	Aw33, B17/Bw58	Southern Chinese	Singapore	[40]
Carbamazepine	SJS/TEN	B*15:02	Han Chinese	Taiwan	[20,35]
			Han Chinese	Hong Kong	[21]
			Thai	Thailand	[23]
			Malaysian	Malaysia	[22]
			Asian	Southeastern countries	[26,41]
			Indian	India	[24]
	SJS/TEN	B*15:11	Japanese	Japan	[34]
	SJS/TEN	B*59:01	Japanese	Japan	[38]
	SJS	B44	Korean	Korea	[38]
			Caucasian	Europe	[26,41]
	MPE/DRESS, DRESS	A*31:01	Han Chinese	Taiwan	[35,42]
			Caucasian	Europe	[28]
			Japanese	Japan	[29,43]
Oxcarbazepine	SJS/TEN	B*15:02, B*15:18	Han Chinese, Taiwanese	Taiwan	[36,44]
Phenytoin	SJS/TEN	B*15:02	Han Chinese, Thai	Hong Kong, Thailand, Taiwan	[5,21,23]
Abacavir	HSS/MPE	B*57:01	Western Australian, Caucasian	Australia, United States	[37,45,46,47]
Nevirapine	DRESS	DRB1*01:01 Cw8-B14	Hispanics, African Caucasian	Africa Italy	[48] [49]
Dapsone	HSS	A*13:01	Han Chinese	China	[33]

CBZ: carbamazepine; DRESS: drug reaction with eosinophilia and systemic symptoms; HSS: hypersensitivity syndrome; SJS: Stevens-Johnson syndrome; MPE: maculopapular exanthema; TEN: toxic epidermal necrolysis.

## Abbreviations

DRESS	drug reaction with eosinophilia and systemic symptoms
HLA	human leukocyte antigen
LTT	lymphocyte transformation test
SCAR	severe cutaneous adverse reaction
SJS	Stevens–Johnson syndrome
TEN	toxic epidermal necrolysis
